# Selected Drug-Likeness Properties of 2-Arylidene-indan-1,3-dione Derivatives—Chemical Compounds with Potential Anti-Cancer Activity

**DOI:** 10.3390/molecules26175256

**Published:** 2021-08-30

**Authors:** Robert Pluskota, Karol Jaroch, Piotr Kośliński, Blanka Ziomkowska, Agnieszka Lewińska, Stefan Kruszewski, Barbara Bojko, Marcin Koba

**Affiliations:** 1 Department of Toxicology and Bromatology, Collegium Medicum im. L. Rydygier in Bydgoszcz, Nicolaus Copernicus University, 87-100 Toruń, Poland; pluskota.r@gmail.com (R.P.); piotr.koslinski@cm.umk.pl (P.K.); 2 Department of Pharmacodynamics and Molecular Pharmacology, Collegium Medicum im. L. Rydygier in Bydgoszcz, Nicolaus Copernicus University, 87-100 Toruń, Poland; karol.jaroch@cm.umk.pl (K.J.); bbojko@cm.umk.pl (B.B.); 3 Department of Biophysics, Collegium Medicum im. L. Rydygier in Bydgoszcz, Nicolaus Copernicus University, 87-100 Toruń, Poland; bziomkowska@gmail.com (B.Z.); skrusz@cm.umk.pl (S.K.); 4 Faculty of Chemistry, University of Wroclaw, Joliot-Curie 14, 50-383 Wroclaw, Poland; agnieszka.lewinska@uwr.edu.pl

**Keywords:** indandione derivatives, lipophilicity, anti-cancer drugs

## Abstract

2-Arylidene-indan-1,3-done derivatives have very different properties, thanks to which they find various applications in science, medicine, and industry. Selected derivatives show antiviral, antibacterial, and anti-inflammatory activity. This paper presents a procedure for the synthesis of a series of indan-1,3-dione derivatives that present antiproliferative activity. The aim of the work was to develop a method of simple synthesis and purification, evaluate the fulfillment of the Lipiński’s and Veber’s rule, and determine the potential scope of application of the obtained series of compounds. The structure of the synthesized compounds was confirmed, and their lipophilicity was determined using experimental and computational methods. Their antiproliferative activity against selected cell lines was tested in accordance with the MTT protocol; the ability to bind to albumin was tested, and the parameters related to the toxicity of substances in silico were determined. The selected compounds which showed antiproliferative activity were strongly bound to albumin and, in most cases, met the Lipiński’s and Veber’s rule. Thus, the obtained results suggest that 2-arylidene-indan-1,3-done derivatives appear to be good candidates for drugs with a potential leading structure for further development.

## 1. Introduction

Indan-1,3-dione derivatives have been of interest to researchers for many years. These compounds have found application in many fields of science. Indandione derivatives have been used as antiplatelet compounds in the treatment of patients with coagulation disorders; they may show a coagulating effect, constitute a derivatizing reagent and a reagent that induces fingerprints. Indandione derivatives can also be used in the treatment of Alzheimer’s disease, treatment of HPV infections, or as an effective rodenticide. Indandione, depending on the substituted groups, has many biological activities and easily undergoes many chemical reactions that enable the preparation of new compounds with industrial or medical properties [1].

The Lipiński’s rule (known as the five-rule or the Pfizer five-rule) allows the assessment of the likelihood that a test chemical with some pharmacological or biological activity is likely to be orally active in humans. This principle was first presented by Christopher A. Lipiński in his research [2,3] and is successfully applied to this day. The Lipiński’s rule is based on a few simple assumptions that are related to the lipophilicity of the tested compound, its molecular weight, and the number of hydrogen bond donors and acceptors (Table 1), while it has some limitations discussed by McKerrowow and Lipiński himself in their article from 2017 [4]. Another set of principles useful in assessing the usefulness of chemical compounds in drug design is the Veber’s rule [5], which applies to other properties of molecules, but is as important as the Lipiński’s rule (Table 1). The application of both is extremely useful in drug design because meeting the conditions of the above-mentioned rules increases the chance of designing a substance that can become a drug candidate.

The term lipophilicity is the affinity of a molecule to the lipid environment (the ability of molecules to dissolve in a hydrophobic environment). This property is extremely important in the drug design process as the pharmacological, pharmacokinetic, and pharmacodynamic activities can be determined by lipophilicity [6].

The molecular properties of chemical structures are the resultant of many factors that determine the similarity of chemical compounds to already known and used drugs. Two of the main reasons for failure in drug development are inadequate pharmacokinetic properties and high toxicity. After introducing the idea of drug-likeness, it became an indispensable element of creating new structures that could be used in the future in general pharmacotherapy [7].

The aim of this study was to perform a series of syntheses of indan-1,3-dione derivatives, which may be candidates for new drugs, and to test the compliance of the tested compounds with the Lipiński’s and Veber’s rules. Additionally, the aim of this study was to compare lipophilicity parameters obtained by various techniques and to select structures that will be directed to further improvement of properties and detailed in vitro tests and possible animal tests.

## 2. Results

### 2.1. Synthesis Mechanism

The proposed method of synthesis allowed us to obtain the expected reaction products with the efficiency allowing for obtaining compounds for the next research stages. The most probable mechanism of synthesis of the indan-1,3-dione derivatives in question is the mechanism based on the aldol condensation mechanism.

**Figure 1:**
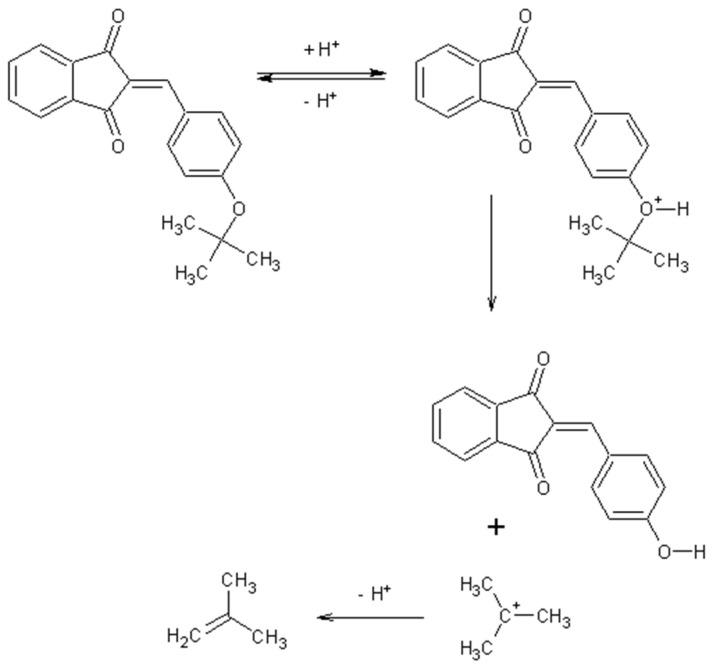
Mechanism of the synthesis reaction of 2-[(4-hydroxyphenyl) methylidene]-1H-indene-1,3 (2H)-dione.

In the case of 3J, we are dealing with an unexpected product. The presence of a hydroxyl group in the para position instead of the expected *tert*-butyloxy group could be caused by a proton attack on the oxygen atom of the *tert*-butyloxy group, which led to the formation of the tertiary carbocation. It is also worth mentioning that the nuclear magnetic resonance did not show the presence of the *tert*-butyloxy group or the transformation products of this group in the tested samples (Figure 1).

### 2.2. Assumptions of the Lipiński’s and Veber’s Rules

The values of lipophilicity determined experimentally using the Snyder–Soczewiński model indicate that most of the tested compounds have logk_w_ values below 5 in analyses using methanol or acetonitrile as organic modifiers, which indicates that these chemicals should freely penetrate biological membranes. The exceptions are compounds described as **1D** and **1I**, which do not meet this basic assumption of the Lipiński’s rule, but only in analyses using methanol as a modifier. Depending on the organic modifier used in the analysis, different results were obtained. For individual indan-1,3-dione derivatives, different values of the Δ parameter were received, which describes the differences in logk_w_ values depending on the modifier used in the mobile phase (Table 2). The coefficient of determination of the regression curves shows an almost complete linear correlation of the obtained data, which clearly indicates the high precision of the chromatographic method in determining lipophilicity.

The lipophilicity values determined by calculation methods are summarized in Table 3. The analysis of this compilation and the lipophilicity parameters obtained experimentally (logk_w-MeOH_ and logk_w-ACN_) distinctly show that there are great differences in the values for the selected compounds, which implicitly represent the same size.

The determined LD_50_ values are shown in Table 4.

**Figure 2:**
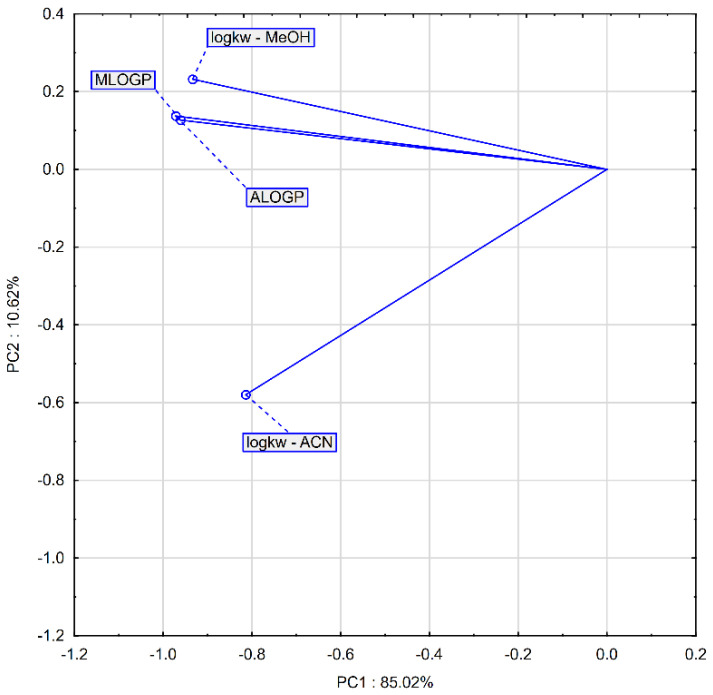
Projection of variables determining lipophilicity on the factor-plane.

**Figure 3:**
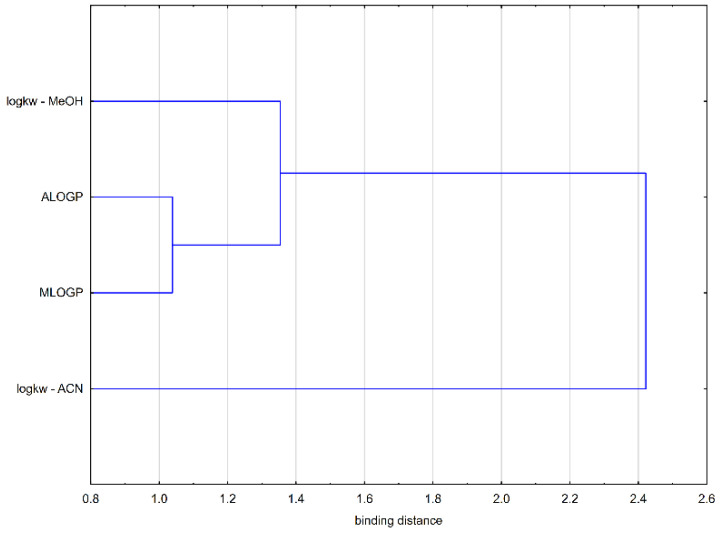
Dendrogram of lipophilicity parameters.

During the principal components analysis (PCA), two main components were distinguished that explain 95.64% of the original variation (PC1: 85.02%, PC2: 10.62%). PCA showed that the values of the lipophilicity parameters obtained by calculation are very strongly correlated with each other. The correlation of logk_w-MeOH_ with the computational parameters is much higher than the correlation with logk_w-ACN_ (Figure 2). Similar observations can be made on the basis of the analysis of the dendrogram (Figure 3).

**Figure 4:**
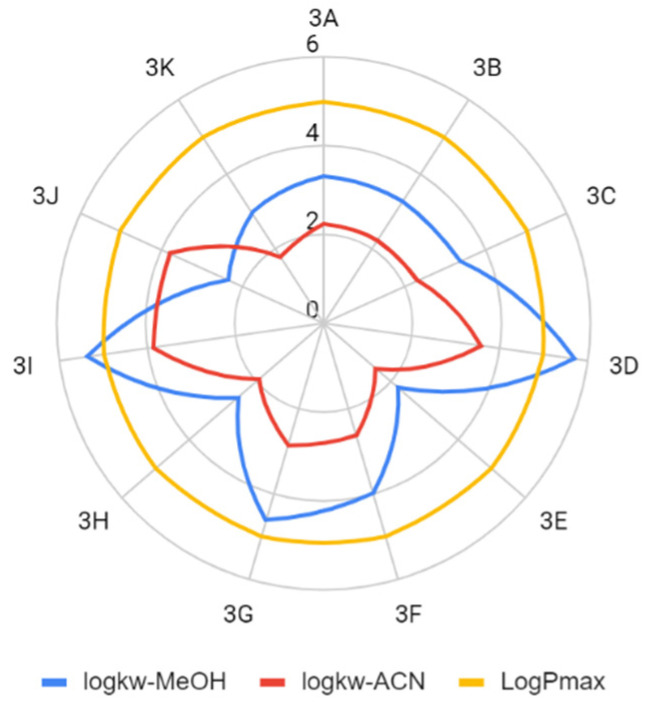
Comparison of the experimental lipophilicity values of the compounds tested (LogPmax—maximum logP value that satisfies Lipiński’s rule).

**Figure 5:**
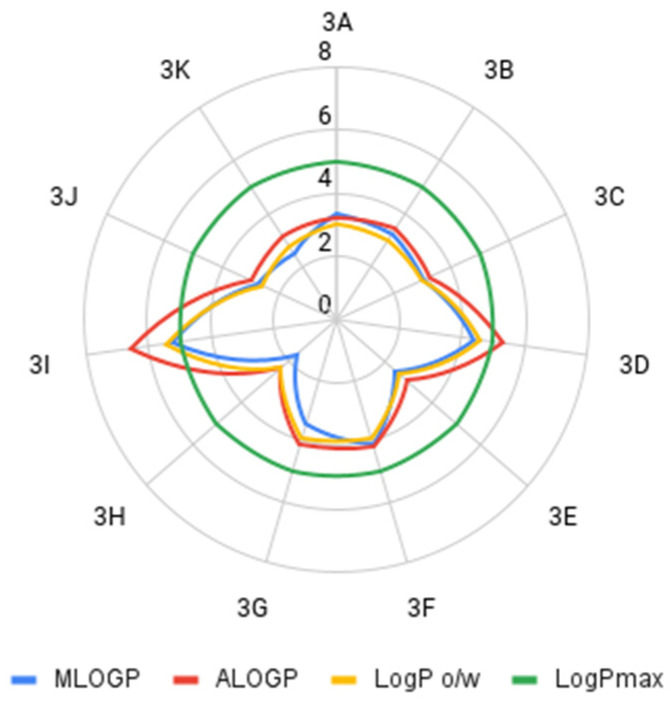
Comparison of the calculated lipophilicity values of the compounds tested (LogPmax—maximum logP value that satisfies Lipiński’s rule).

**Figure 6:**
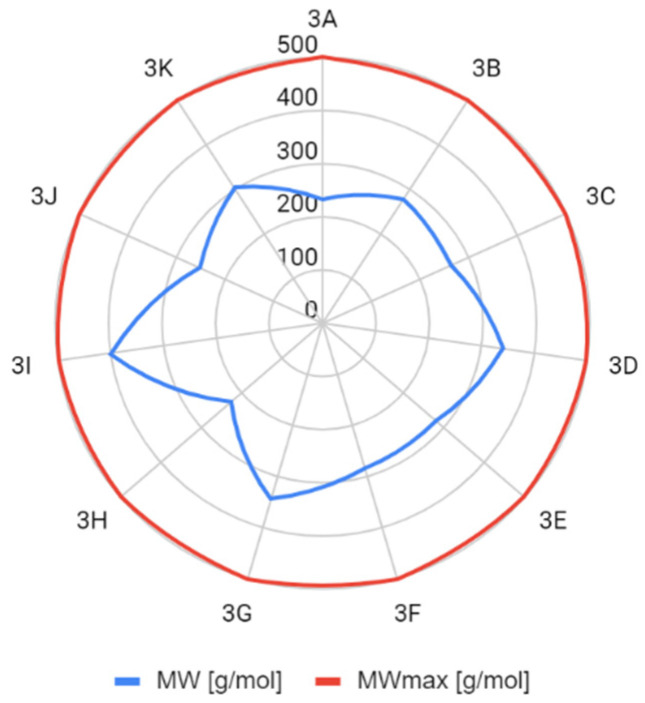
Molecular weight comparison (MWmax—maximum molecular weight value that satisfies Lipiński’s rule).

**Figure 7:**
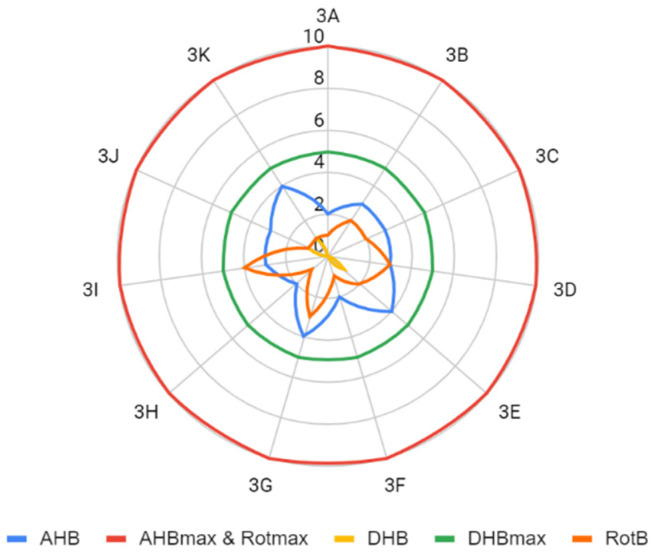
Comparison of the number of bonds (AHB—number of hydrogen bond acceptor, AHBmax—maximum number of hydrogen bond acceptor that satisfies Lipiński’s rule, Rotmax—maximum number of rotating bonds that satisfies Veber’s rule, DHB—number of hydrogen bond donors, DHBmax—maximum number of hydrogen bond donors that satisfies Veber’s rule, RotB—number of rotating bonds).

**Figure 8:**
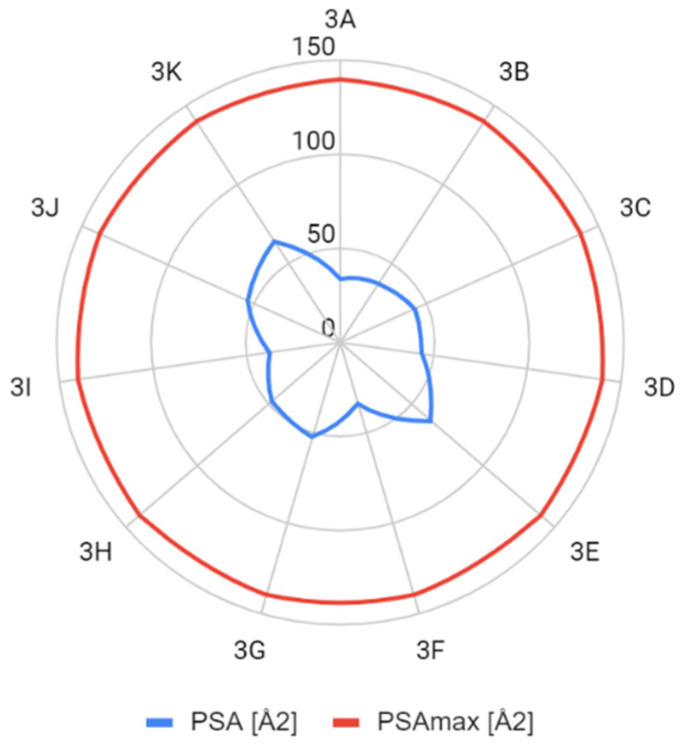
Comparison of polar surface area (PSAmax—maximal polar surface area that satisfies Veber’s rule).

The determined parameters for the Lipiński’s and Veber’s rules were visualized and evaluated. In terms of molecular weight, the number of hydrogen bond donors and acceptors, the number of rotating bonds, and the total polar area, the obtained compounds met the Lipiński’s and Veber’s rule (Figure 4, Figure 5, Figure 6, Figure 7 and Figure 8).

In most cases, the compliance with the lipophilicity principle for the Lipiński’s rule was met. The decisive factor in this situation is which lipophilicity parameter we use. When using the experimental method consisting in the determination of the logk_w_ parameter, the obtained result depends on the organic modifier used for the mobile phase, which may lead to different conclusions. For example, logk_w-MeOH_ indicates that compound **3D** will not be orally biologically active. On the other hand, logk_w-ACN_ indicates that the Lipiński’s rule in this respect is fulfilled.

### 2.3. Biological Activity

**Figure 9:**
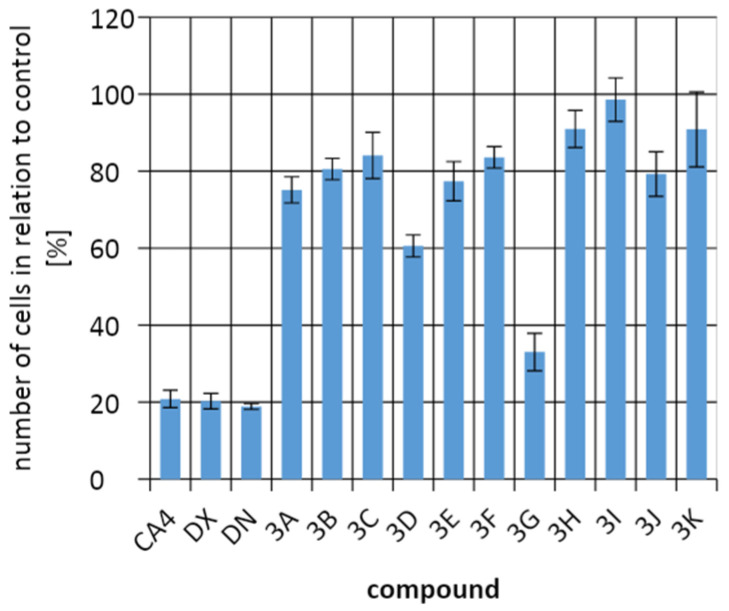
Comparing the number of cells from line A549 in relation to the control culture.

**Figure 10:**
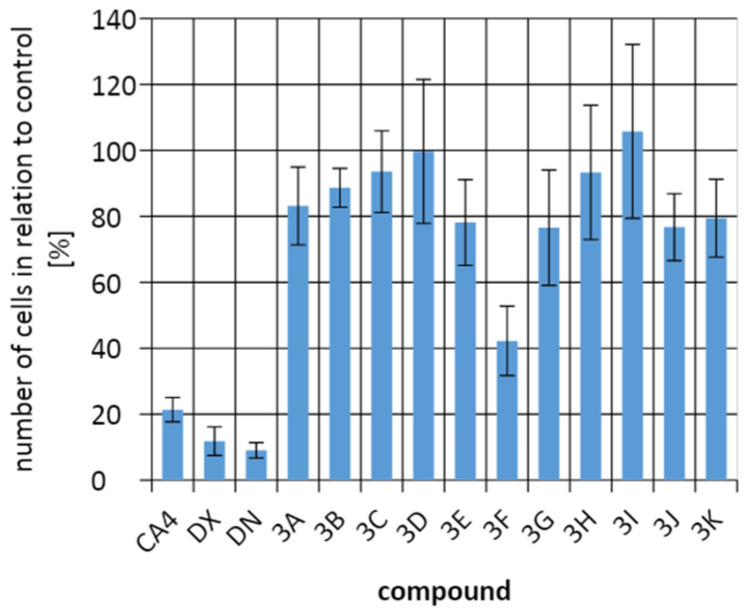
Comparing the number of cells from line B16F10 in relation to the control culture.

**Figure 11:**
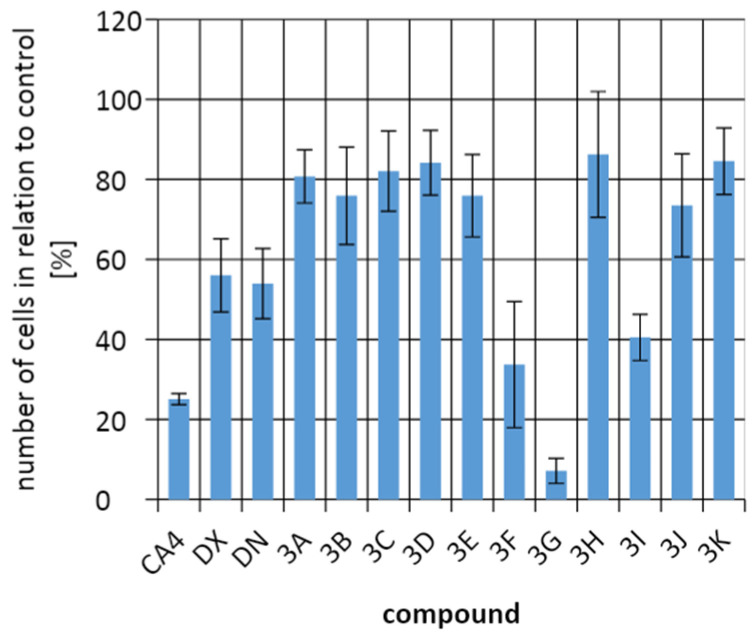
Comparing the number of cells from line HeLa in relation to the control culture.

To evaluate the efficacy of the test compounds on tumor cells, a comparison was made of the percentage of cells in the culture with that in the control culture without the addition of test compounds. The development and division of A549 cells were most strongly inhibited by **3G** and **3D**, but they were still less effective in killing tumor cells than combretastatin 4 (CA4), doxorubicin (DX), and daunorubicin (DN). The B16F10 cells line responded best to control compounds and to compound **3F**. In contrast, for the HeLa cells line, **3G** was more effective than the control compounds. It is important that all compounds had an antiproliferative effect on cells, reducing the number of cancer cells in cultures (Figure 9, Figure 10 and Figure 11).

### 2.4. Binding to Albumin

**Figure 12:**
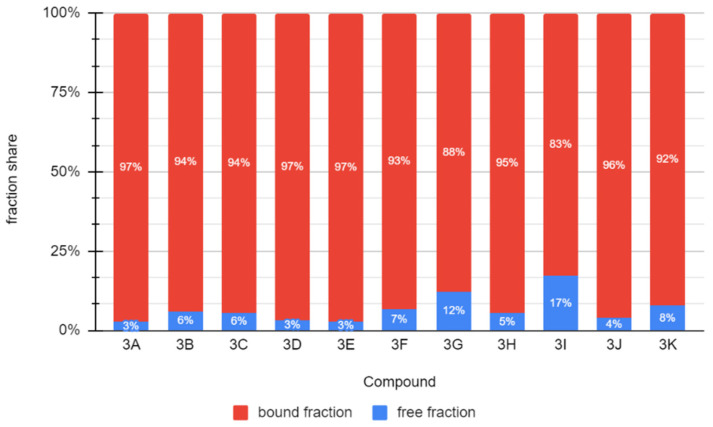
Share of the free fraction of the tested compounds and bound to albumin (for the concentration of albumin in the blood of 44 mg/mL (660 μM)).

The chemical compounds described in this study can bind very well with albumin. The free fraction for 2-substituted indan-1,3-dione derivatives ranges from 3 to 17%, which suggests that these compounds will exhibit prolonged action after administration. These combinations in the body will probably be slowly released from the complex with albumin, which will lead to a relatively low concentration (Figure 12).

### 2.5. Toxicity

In silico acute toxicity studies have shown that the products of the resulting synthesis are less toxic than many routinely used cytostatic drugs. All of the analyzed structures were classified into the category of unclassified or harmful compounds, which is related to their low acute toxicity. Importantly, in silico tests have shown that some synthesis products can cross the blood–brain barrier and at the same time do not show carcinogenic and mutagenic effects (Table 5 and Table 6).

## 3. Discussion

The presented method of synthesis of the compounds discussed in this paper is extremely simple, as it does not require heating—it takes place at room temperature. During the reaction, the product formed spontaneously precipitates out of the reaction mixture, and filtration and recrystallization are enough to isolate it. The proposed method also runs with satisfactory efficiency without the use of specially dedicated catalysts. The use of acetic acid as a solvent ensures the relatively high solubility of the substrates and the possibility of simple removal of the solvent after the synthesis is completed. At present, other techniques have been used to obtain 2-substituted indan-1,3-dione derivatives, whose structure resembles those presented above. Tugrak et al. [11] presented the reaction of indan-1,3-dione with aromatic aldehyde in the presence of sodium hydroxide in ethanol, and the reaction was carried out at an elevated temperature. In 2018, Mitka et al. [12] obtained several compounds according to the method presented in this study (**3A**, **3C**, and **3F**), with the difference that recrystallization from *n*-octane was performed. The remaining compounds have not been prepared by the presented method so far, and the compounds **3D**, **3G**, and **3K** have been synthesized for the first time.

The Snyder–Soczewiński curve is a chromatographic interpretation of lipophilicity and is equivalent to the classical measurement of this parameter. The assumptions of the chromatographic method are simple: a multicratic reverse-phase elution should be carried out with the use of water and the selected organic modifier (usually methanol, acetonitrile, or isopropanol). The retention times obtained in this way are converted into retention coefficients and used to plot their dependence on the share of the organic modifier in a given analysis. The use of various organic modifiers is optional, but it leads to the determination of different logk_w_ values, which will indirectly affect the decisions made by the researcher. If we assume the gold standard of examining the assumptions of the Lipiński’s rule at an early stage of designing a new active substance, the fulfillment of the assumption that lipophilicity should be <5 will depend on the type of modifier used. Therefore, the selection of the measurement method has an impact on the obtained result and leads to different conclusions.

Against selected cell lines, none of the compounds shows antiproliferative activity. The mechanism of this interaction is not fully understood. There are indications in the literature that suggest the mechanism of action of the obtained derivatives. The current publication reports that hydrogen bonds appearing between the amino acids and the carbonyl groups of the indandione are responsible for the interactions with proteins that are part of the cells [13]. The cytotoxic effect is also attributed to the possible interaction of indandione derivatives with the caspase 3 receptor. Moreover, the synthesized derivatives could also act as covalent inhibitors due to the carbonyl alpha, beta-unsaturated motif presented in all compounds [14].

In this work, methanol and acetonitrile were used as organic modifiers during the chromatographic study. After plotting the Snyder–Soczewiński curves and determining the logk_w_ parameter, it turned out that its values differ depending on the modifier used, and they are lower when there is acetonitrile in the liquid phase. Acetonitrile has a higher elution power, which is why it is considered a more efficient modifier, which indirectly influences the lipophilicity value set by chromatography [15]. Accordingly, the use of acetonitrile to determine the logk_w_ parameter by chromatographic method for non-polar compounds will lead to a lower value of this parameter in relation to the measurement with methanol, ethanol, or isopropanol.

The substance **3H**, which is the only one not having a phenyl group but a furyl group, has a very low value of logk_w_. This moiety, being more hydrophilic than the phenyl groups, lowers the lipophilicity of the entire molecule in relation to the others. As the number of phenyl groups increases, the lipophilicity of the compounds increases. The substance **3E** is the most polar when using both methanol and acetonitrile. Particular attention should be paid to compound **3J**, which is the only one that shows a higher logk_w_ value when eluted with acetonitrile. This may be due to the fact that the phenyl group attached to the 2-benzylideneindan-1,3-dione core is strongly protonated, which increases the lipophilicity value of acetonitrile [16], which confirms the thesis presented earlier. Compounds **3A** and **3C** have an additional -OCH_3_ substituent, as do **3F** and **3G** (two such substituents in **3G**). Additional methoxy groups increase the lipophilicity of the compounds. Regardless of the aromatic substituents in the 2-position of the indan-1,3-dione, the tested compounds show activity against the examined structures. 

The determined theoretical parameters of lipophilicity in this study were ALOGP and MLOGP. Determining their values is mainly based on the assumption that the hydrophobicity of substances is nothing else than the difference of free energy in the non-polar and polar phase, which changes under the influence of (1) the enthalpy of interaction between the dissolved molecules and the solvent, (2) the enthalpy of changes occurring between the dissolved molecules, and (3) entropy changes caused by changes in the solvent around dissolved molecules. Every solute–solvent interaction is caused by molecular forces.

The most important properties influencing the solubility of substances are the ability to create hydrogen bonds (increase in hydrophilicity) and the composition of atoms and bonds between them. The ALOGP parameter is based on the hydrophobicity values of individual atoms. The differences in the availability of atoms result from the distribution of electrons around the nucleus and the availability of the solvent for the atom [9]. The MLOGP parameter is additionally based on the topology of the molecule, including the number of carbon atoms, halides, nitrogen, oxygen, unsaturated bonds, and rings [8]. After applying the above methods on the tested compounds, it turns out that the values obtained with both ways are not identical. However, there is some correlation between them—the compounds of **3D**, **3F**, **3G**, and **3I** show the highest lipophilicity, which results from the presence of more than one phenyl group in the substituent. The presence of rings is a factor increasing lipophilicity in both methods [17].

The highest value is observed in a compound having three rings in the substituent. **3D** and **3G** compounds contain ether bonds that increase theoretical lipophilicity, but the main factor increasing their lipophilicity is the number of carbon atoms. The additional phenyl group present in the compound **3D** increases its solubility in non-polar solvents. The lowest lipophilicity occurs in compounds marked with numbers **3E**, **3H**, **3J**, and **3K**. Substance **3H** does not have a phenyl group in the substituent but has a furan group, which is associated with a smaller number of carbon and hydrogen atoms. In the MLOGP method, lipophilicity is additionally lowered by the mere presence of a non-phenolic ring. All other test compounds have a phenyl group in the substituent and differ in the groups attached to it. The hydroxyl group present in compounds **3E** and **3J** increases the hydrophilicity of the compounds; their ALOGP values are very similar, but in the MLOGP method, the position of the functional group is also important. This group is in **3E** and **3J** in *meta* and *para* positions, respectively. The presence of ether bonds has a greater impact on the MLOGP parameter [8].

It is noteworthy that the ALOGP and MLOGP parameters are the most similar for all compounds with logk_w-MeOH_ and are more strongly correlated with it than with logk_w-ACN_. It seems possible to use the experimental parameter logk_w-MeOH_ interchangeably with ALOGP and MLOGP. On the other hand, Kosmalski et al. [18] obtained completely different results in terms of the correlation of the calculation parameters of lipophilicity and the experimental parameters for 1-(benzofuran-2-yl) ethan-1-one oxime and its substituted O-benzyl ethers. It was shown in this work that it is logk_w-ACN_ that is better correlated with the calculation parameters.

In view of the above, it can be assumed that for highly hydrophobic compounds, logk_w-ACN_ better reflects the actual lipophilicity than in the case of the analysis of hydrophilic compounds. The use of the chromatographic method of lipophilicity determination is burdened with an incorrect selection of the organic modifier, which leads to the determination of a parameter significantly different from the actual lipophilicity value. However, this does not pose a great threat to the conduct of research, as there are numerous computational alternatives for experimental parameters more favorable in terms of the time to obtain the result and the price of the analysis.

The discussed compounds were not indifferent to all tested cell lines. On average, no more cells were observed in any of the cultures than in the control culture. The tested compounds showed the strongest effect on HeLa cells. Against tumor cells of the A549 line, the control compounds and compound **3G** showed the strongest effect. In the case of the B16F10 cells line, it was the control compounds and the compound **3F** that inhibited the cell division the most. The best results were obtained for HeLa cells, where the **3G** compound showed the best activity. Incubation of HeLa cells with **3G** allowed to obtain only 7.15 ± 3.12% of cells compared to the control culture. The next place in the number of cells for the HeLa line was taken by CA4, followed by compounds **3F** and **3I**.

**3F** and **3G** have the greatest potential in terms of the influence of the tested compounds on selected tumor cell lines. Additionally, it is worth mentioning that both compounds meet the assumptions of the Lipiński’s and Veber’s rules and their LD_50_ values are relatively high. It is noteworthy that the **3G** compound most likely has the ability to cross the blood–brain barrier, which may be useful in the further stages of work on searching for its application. Unfortunately, both lead structures may have mutagenic properties.

Ligand-lipophilicity efficiency (LLE) is a parameter derived from the lipophilicity value and the half-maximal inhibitory concentration (IC_50_) or the half-maximal effective concentration (EC_50_). LLE is used successfully in the drug design process to assess the quality of test compounds, linking potency and lipophilicity to assess drug-likeness. In the screening of new chemicals for biological activity, LLE is used to select a series of chemicals for further optimization [19]. To calculate the LLE value, it is necessary to determine the lipophilicity. For this purpose, mainly calculation methods are used. For IC_50_ and EC_50_, it is necessary to perform experiments if no predictive models are available to predict biological activity. In this study, at the screening stage of a new series of indan-1,3-dione derivatives, the IC_50_ value was not determined, as the determination of the number of tumor cells after incubation with the obtained derivatives in relation to the control culture was sufficient to select potentially useful compounds for the further design. It is worth mentioning, however, that the optimal value of lipophilicity should be in the range of 2–3, which is a compromise between water and lipid solubility [20]. In view of the above, more than 60% of the compounds described in the present study are within the optimal lipophilicity range.

## 4. Materials and Methods

The synthesis was performed according to the procedure described in the experimental part. Then, the obtained compounds were separated from the reaction mixture and purified; their structures were confirmed using nuclear magnetic resonance and mass spectrometry and further analyzed.

The obtained products were subjected to chromatographic analysis using the RP-HPLC technique in order to determine the lipophilicity of the obtained compounds, to assess whether the synthesized molecules have a chance to be biologically active after oral administration, and to test whether the obtained compounds meet the Lipiński’s and Veber’s rules.

The antiproliferative activity was tested according to the MTT protocol.

The binding capacity to albumin was also determined, and the toxicity class was determined using in silico analyses. The geometry of the obtained structures was also optimized, and the lipophilicity parameters were determined using computational methods.

### 4.1. Synthesis, Purification, and Structure Conformation—General Procedure

The synthesis of compounds in this study was based on the reaction of indan-1,3-dione and the appropriate aromatic aldehyde according to the following scheme in the table (Figure 1, Appendix A). An equimolar amount of substrates was used, and the reactions were carried out at room temperature for 72 h in concentrated acetic acid with the addition of a catalytic amount of concentrated sulfuric acid (VI) with constant stirring. The product that precipitated out of solution was filtered off and washed with concentrated acetic acid and then recrystallized from n-hexane. The finished product was then dried under reduced pressure at −78 °C and pressure of 0.0001 mbar. Nuclear magnetic resonance and mass spectrometry were used to confirm the structure of the compounds. Indan-1,3-dione and aldehydes, in addition to benzaldehyde, were purchased from Sigma Aldrich (Poznań, Poland). Acetic acid, sulfuric acid, benzaldehyde, and *n*-hexane were purchased from Avantor (Gliwice, Poland). The product was weighed, and the yield was calculated. Compounds were identified using an AVANCE III NMR 500 MHz spectrometer from Brucker Co. (Billerica, MA, USA) and a Shimadzu LCMS-8040 system (Kyoto, Japan).

### 4.2. Chromatographic Examination of Lipophilicity

The chromatographic analysis was carried out on a Shimadzu device equipped with a pump system (LC-20AD model), a detector with a diode bench (SPD-M20A model), a degasser (DGU-20A 5R model), a chromatographic oven (CTO-10AS VP), autosampler (SIL-20AC HT model), and chromatographic column (LiChrospher 100 RP-18, 5 μm, Poznań, Poland). Analyses were performed, leading to a multicratic elution. The mobile phase was methanol (J. T. Backer, Phillipsburg, NJ, USA) or acetonitrile (J. T. Backer) and water (Hydrolab, HPL5UV model, Straszyn, Poland) in different volume ratios. In individual analyses, the concentration of the organic modifier expressed as a mole fraction was 0.75–0.95 with a constant step of 0.05. The test compounds were dissolved in methanol. The injection volume was 10 µL. The flow was set at 1 mL/min, the temperature of the chromatography oven at 25 °C, and the detector temperature at 2 °C higher than the ambient temperature. Compounds were detected in the range 190–800 nm. All samples were tested in duplicate at least. Column dead time (*t_M_*) was determined using uracil (Sigma Aldrich). The retention coefficient was calculated using the following formula:

(1) $$k=\frac{t_{R}}{t_{M}}-1,$$

where *t_R_* is the retention time of the test compound in a given eluent composition, and *t_M_* is the chromatographic column dead time.

The value of lipophilicity was determined using the linear dependence of the decimal logarithm from the retention coefficient (logk) and the concentration of the organic modifier in the mobile phase, which was expressed as a volume fraction. For this purpose, the following formula was used:

(2) $$logk=logk_{w}+S_{\varphi },$$

where *logk* is the decimal logarithm of the retention coefficient, *logk_w_* is the value obtained by extrapolating the curve, *S* is the slope of the regression curve, and *φ* is the volume fraction of the organic modifier.

The coefficient of determination (*R*^2^) was also calculated for the prepared curves.

### 4.3. Geometry Optimization of Structures

The particle geometry was optimized with the use of the HyperChem 8 (Hypercube, Gainesville, FL, USA) program with the use of the MM + force field in accordance with the principles of quantum mechanics (initial optimization) and the semi-empirical method AM1 and the Polak-Ribiere algorithm (proper optimization). The iteration limit was set at 50, and the value of the potential gradient used in the calculations was 0.01 kcal/(Å⋅mol). The maximum number of cycles was 32,000.

The optimized structure was used to determine parameters related to the Veber’s and Lipiński’s rule in the Dragon software (Talete, Milan, Italy).

### 4.4. Testing of Antiproliferative Activity

Mouse melanoma (B16F10), human lung carcinoma (A549), and human cervix (HeLa) cell lines (kindly gifted by Prof. Tomasz Drewa, a Chair of Urology, Department of Regenerative Medicine, Collegium Medicum, Nicolaus Copernicus University, Bydgoszcz, Poland) were cultivated at 37 °C, controlled CO_2_ atmosphere (Binder, Tuttlingen, Germany), and high humidity with RPMI cell culture medium (with L-glutamine and sodium bicarbonate, Sigma-Aldrich), supplemented with 10% of fetal bovine serum (FBS, Biowest, Nuaillé, France), as well as antibiotics (penicillin, streptomycin) and antimycotics (amphotericin B) (Sigma-Aldrich). Combretastatin A4 (CA4), doxorubicin (DoX), and doxorubicin (DN) were purchased from Sigma-Aldrich.

Cells were counted with an automated cell counter (Countess^®^ II FL, Invitrogen by Thermo Fisher Scientific Inc., Waltham, MA, USA). A number of 1 × 10^3^ (B16F10, A549) or 2.5 × 10^3^ cells were plated in each well of 96-well plate (Nunc Edge 2F, Thermo Fisher Scientific Inc.) and left for 24 h incubation prior to drug administration. After this period, the medium was replaced with a fresh medium containing the drugs (at final concentration of 0.01 mg/mL) and left for incubation for the next 72 h. A solution of thiazol blue tetrazolium (MTT, Sigma-Aldrich) was prepared in PBS at concentration of 5 mg/mL and administrated to the cell culture medium at the volume of 10% of the initial medium volume. After 2 h of incubation, the medium was removed, and crystals of formazan were solubilized in isopropanol (Sigma-Aldrich). Absorbance readout was conducted using plate reader Multiskan Spectrum (Thermo Electron Corporation, Waltham, MA, USA) at 570 nm.

Combretastatin A4 (CA4), doxorubicin (DX), and doxorubicin (DN) were employed as positive controls. Biological evaluation was performed as three independent experiments; mean response and standard deviation were calculated using Excel software (v. 2013, Microsoft Corporation, Redmond, WA, USA).

### 4.5. Measurement of the Ability to Bind to Albumin

Fluorescence lifetime and fluorescence intensity of HSA solutions were measured using the time-resolved Fluorescence Spectrophotometer Life Spec II (Edinburgh Instruments Ltd., Livingston, UK) with the sub-nanosecond pulsed EPLED diode emitting a light of the wavelength 280 nm. The spectrometer was equipped with electronically cooled photomultiplier Hamamatsu R928 connected with TCC900 PC Card, which incorporates all the electronic modules required for Time-Correlated Single Photon Counting (TCSPC). The fluorescence decays were recorded at an emission wavelength of 350 nm. The data were fitted with an exponential decay to obtain total fluorescence intensity and lifetime. Measurements were performed at room temperature using quartz cuvettes 3.5 × 10 mm. The exposure time of the sample was 30 s. A spectrophotometer Jasco V-550 was used for absorption measurements.

Human Serum Albumin (HSA, 97%) was purchased from Sigma Aldrich. For the fluorescence quenching measurements, the 10 µM PBS solution of HSA at pH 7.4 was prepared, and 10 mM stock solutions of studied compounds were prepared in DMSO (dimethylsulfoxide). The final concentration of studied compounds in HSA solution varied between 0 and 70 µM.

Fluorescence quenching of albumin can be described by the Stern–Volmer Equation [21]:

(3) $$\frac{F_{0}}{F}=K_{SV}[Q]+1,$$

where *F*_0_ and *F* are the fluorescence intensities in the absence and presence of quencher, respectively, [*Q*] is the concentration of quencher, and *K_SV_* is the Stern–Volmer quenching constant.

Due to the fact that a quencher can absorb light at the excitation and/or emission wavelengths, the measured intensity should be corrected according to the Formula [22]:

(4) $$F_{observed}=F_{correct}\times 10^{-\frac{A_{ex}\times d_{ex}}{2}-\frac{A_{em}\times d_{em}}{2}},$$

where *F_observed_* is the measured fluorescence, *F_correct_* is the fluorescence that would be measured in the absence of absorption effect, *A_ex_* and *A_em_* are the absorbances of ligand at the excitation (280 nm) and emission wavelength (350 nm), respectively, and *d_ex_* and *d_em_* are the cuvette pathlength in the excitation and emission direction (0.35 cm and 1 cm, respectively).

As the fluorophore can be quenched by collisions or by complex formation with the quencher, there are two types of quenching: dynamic and static. They can be distinguished by lifetime measurements. In dynamic quenching, the decrease in yield and lifetime of fluorescence occurs, while static quenching does not decrease the lifetime. If the quenching is known to be dynamic, then:

(5) $$\frac{F_{0}}{F}=K_{D}[Q]+1=\frac{\tau_{0}}{\tau },$$

where *τ*_0_ and *τ* are the lifetimes in the absence and presence of quencher, and *K_D_* is the dynamic quenching constant.

In many cases, both static and dynamic quenching occurs for the same fluorophore, then modified Stern–Volmer equation has the form:

(6) $$\frac{F_{0}}{F}=(K_{D}[Q]+1)(K_{S}[Q]+1)=\frac{\tau_{0}}{\tau }(K_{S}[Q]+1),$$

The dynamic portion of the quenching (*K_D_*[*Q*] + 1), according to Equation (5), can be determined by lifetime measurements. The portion (*K_S_*[*Q*] + 1) relates to static quenching, and K_S_ is the association constant, which is given by [21]:

(7) $$K_{S}=\frac{\left[F-Q\right]}{\left[F][Q\right]},$$

where [*F*−*Q*] is the concentration of complex fluorophore–quencher, [*F*] is the concentration of uncomplexed fluorophore (concentration of free HSA molecules), and [*Q*] is the concentration of quencher.

In this study, the tested compounds were added to the HSA solution, and the fluorescence intensity and lifetime at 350 nm were measured for varying concentrations of ligands. On the basis of absorbance measurements of ligands at 280 nm and 350 nm, the correct fluorescence intensities were estimated using Equation (4). Then, the Stern–Volmer constants, dynamic quenching constants, and association constants of studied compounds were determined using Equations (3), (5) and (6), respectively.

Knowing the value of association constant *K_S_*, it is possible to determine the theoretical percentage of the bound and free fraction of the drug for the physiological concentration of albumin. Assuming that the concentration of albumin [HSA] is much higher than the concentration of the drug and that the binary complex is formed, the percentage of bound [*C_B_*_%_] and free fraction [*C_F_*_%_] of the drug can be calculated according to the following Formula [23]:

(8) $$\left[C_{B\%}\right]=\frac{\left[HSA\right]}{[HSA]+1/K_{S}}\times 100\%,$$

(9) $$\left[C_{F\%}\right]=100\%-\left[C_{B\%}\right],$$

In this study, the blood albumin concentration [HSA] was assumed to be 660 µM.

### 4.6. Toxicity Simulation

Using the GUSAR Online tool [24], the LD_50_ value was predicted based on the structure of the molecule. Indan-1,3-dione and doxorubicin were used as comparative compounds. Using the Lazar Toxicity Predictions online tool [25], the assessment of mutagenicity (in *Salmonella typhimurium*), carcinogenicity (in rats), ability to cross the blood–brain barrier (in humans), and acute toxicity (in *Fathead minnow* and *Daphnia magna*) was performed.

### 4.7. Statistical Analysis

The obtained data were statistically analyzed using the Statistica 13.3 statistical package (TIBCO Software Inc, Palo Alto, CA, USA). Principal component analysis (PCA) was performed using the covariance matrix. The scree plot and the Kaiser criterion were used to distinguish the number of factors. In contrast, when compiling the dendrogram, the Euclidean distance was used as a measure of the distance between variables and the single-link method to define the distance between clusters. All analyses were performed at the significance level of 5% (α = 0.05), and the input data were previously standardized.

## 5. Conclusions

The selected chemical compounds discussed in this study seem to be sufficiently good candidates for drugs in terms of their structure, lipophilicity, toxicity, and biological activity. The performed analyses show that most likely, these compounds may be biologically active after oral administration because the tested molecules dissolve well enough in the hydrophobic environment that cell membranes will not constitute a barrier for them.

2-[(4,7-dimethoxynaphthalen-1-yl)methylidene]-1H-indene-1,3(2H)-dione (**3G**) can be defined as the best leading structure for cervical cancer antitumor activity for the development of the target active and finished substance formulation for the next stages of research.

The logk_w_ parameter has different values for the different organic modifiers used. Therefore, an important factor influencing the interpretation of the usefulness of compounds as drugs is the organic modifier used in the chromatographic analysis. The type of organic modifier used has a key impact when interpreting the values of lipophilicity parameters based on the Lipiński’s rule.

## Figures and Tables

**Table 1:** Conditions included in Lipiński’s rule and Veber’s rule characterizing substances that are good candidates for drugs.

Lipiński’s Rule		Veber’s Rule	
Feature	Value	Feature	Value
LogP	<5	number of rotating bonds (RotB)	≤10
molecular weight (MW)	<500 Da		
number of hydrogen bond acceptors (AHB)	<10	polar surface area (PSA)	<140 Å^2^
number of hydrogen bond donors (DHB)	<5		

**Table 2:** The values of lipophilicity determined by the experimental method and the values of parameters describing the Snyder–Soczewiński curve.

Compound	MeOH			ACN			Δ *
	logk_w_	R^2^	−S	logk_w_	R^2^	−S	
**3A**	3.3210	0.9907	3.3278	2.2433	0.9974	2.4449	1.0777
**3B**	3.2790	0.9955	3.1597	2.2394	0.9982	2.3613	1.0396
**3C**	3.3554	0.9968	3.2782	2.3042	0.9976	2.4879	1.0512
**3D**	5.6763	0.9966	5.4798	3.5726	0.9993	3.5570	2.1037
**3E**	2.2321	0.9917	2.4114	1.5356	0.9919	1.9201	0.6965
**3F**	3.9640	0.9967	3.8549	2.6107	0.9987	2.7449	1.3533
**3G**	4.6064	0.9972	4.6064	2.8533	0.9991	2.8762	1.7531
**3H**	2.5521	0.9959	2.6243	1.9162	0.9950	2.2011	0.6359
**3I**	5.3524	0.9994	5.0483	3.8489	0.9995	3.7589	1.5035
**3J**	2.3814	0.9952	2.5802	3.7694	0.9997	3.7694	1.3880
**3K**	2.9717	0.9946	3.0667	1.7720	0.9967	2.1213	1.1997

* Δ = |logk_w-MeOH_ − logk_w-ACN_|.

**Table 3:** Theoretical parameters of lipophilicity for tested compounds.

Compound	MLOGP *	ALOGP **	LogP o/w ***
**3A**	3.322	3.236	3
**3B**	3.235	3.399	3
**3C**	2.998	3.22	2.99
**3D**	4.402	5.283	4.6
**3E**	2.458	2.953	2.61
**3F**	4.116	4.145	3.91
**3G**	3.451	4.112	3.89
**3H**	1.664	2.34	2.31
**3I**	5.218	6.569	5.45
**3J**	2.754	2.969	2.58
**3K**	2.462	3.134	2.76

* MLOGP—atomic octanol/water partition coefficient [8]; ** ALOGP—atomic octanol/water partition coefficient [9]; *** LogP o/w—average LogP value obtained using the SwissADME tool [10].

**Table 4:** In silico LD_50_ values are determined and the compounds are classified according to their acute toxicity.

Compound	LD_50_ *	Toxicity Class **
**3A**	1113.0	harmful
**3B**	1707.0	harmful
**3C**	3167.0	unclassified
**3D**	2937.0	unclassified
**3E**	2608.0	unclassified
**3F**	742.5	harmful
**3G**	1707.0	harmful
**3H**	1264.0	harmful
**3I**	2176.0	unclassified
**3J**	2373.0	unclassified
**3K**	577.4	harmful
indan-1,3-dione	297.8	harmful
doxorubicin	110.8	toxic

* Lethal dose causing death of half of the studied rat population after oral administration (mg/kg bw). ** Classification of the oral toxicity of a chemical used in the European community.

**Table 5:** Comparison of acute toxicity values and the ability of test compounds to overcome the blood–brain barrier, carcinogenicity, and mutagenicity.

Compound	Acute Toxicity [mg/L]		Overcoming the Blood–Brain Barrier *	Carcinogenicity **	Mutagenicity ***
	*Fathead minnow*	*Daphnia magna*			
**3A**	0.236	6.58	penetrating	non-carcinogenic	mutagenic
**3B**	21.4	9.65	non-penetrating	carcinogenic	non-mutagenic
**3C**	32.1	3.59	non-penetrating	non-carcinogenic	mutagenic
**3D**	69.8	42.2	penetrating	non-carcinogenic	mutagenic
**3E**	31.7	9.4	non-penetrating	non-carcinogenic	mutagenic
**3F**	4.51	2.82	non-penetrating	non-carcinogenic	mutagenic
**3G**	64.5	9.3	penetrating	carcinogenic	mutagenic
**3H**	- ****	- ****	penetrating	non-carcinogenic	non-mutagenic
**3I**	- ****	10.6	non-penetrating	non-carcinogenic	mutagenic
**3J**	13.7	12.3	penetrating	non-carcinogenic	mutagenic
**3K**	24.4	23.4	non-penetrating	non-carcinogenic	mutagenic
indan-1,3-dione	52.9	25.7	penetrating	non-carcinogenic	non-mutagenic
Doxorubicin	- *****	- *****	non-penetrating	carcinogenic	mutagenic

* simulation for a human. ** simulation for a rat. *** simulation for *Salmonella typhimurium*. **** Cannot create prediction: only one similar compound in the training set. ***** Could not find similar substances with experimental data in the training dataset.

**Table 6:** General synthesis procedure.

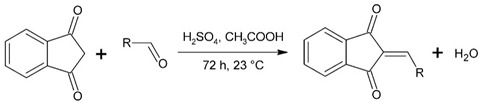		
**1** **2A**–**2K** **3A**–**3K** **4**		
** 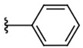 **	** 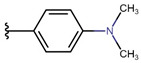 **	** 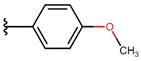 **
**A**	**B**	**C**
** 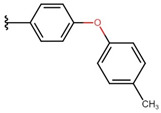 **	** 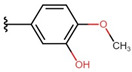 **	** 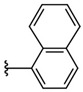 **
**D**	**E**	**F**
** 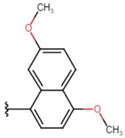 **	** 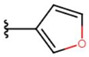 **	** 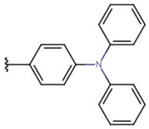 **
**G**	**H**	**I**
** 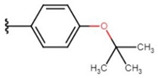 **	** 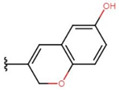 **	
substrate		
** 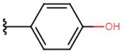 **		
product		
**J** *	**K**	

* an unexpected synthesis product.

## Data Availability

All data obtained during the research appear in the submitted article.

## Sample Availability

Samples of the indandione derivatives compounds are available from the authors.

## Appendix A

Detailed synthesis procedure and confirmation of the structure of the tested compounds.

2-benzylidene-1H-indene-1,3(2H)-dione (**3A**)
  The product was obtained as a yellow powder; 0.645 g (27.53%). m.p. 152–153 °C. C_16_H_10_O_2_ (calcd 234.26). ESI-MS: (M + H)^+^ = 235.10. ^1^H-NMR (CDCl_3_), δ [ppm]: δ 8.45 (m, 2H -Ar), 8.00 (m, 2H -Ar), 7.89 (s, 1H =CH-), 7.80 (m, 2H -Ar), 7.53 (m, 3H -Ar). ^13^C-NMR (CDCl_3_), δ [ppm]: 190.43 (aliphatic C in ring), 189.18 (aliphatic C in ring), 147.14 (=CH-), 142.71, 140.24, 135.57, 134.30, 133.34, 129.35, 129.00, 123.50.
2-{[4-(dimethylamino)phenyl]methylidene}-1H-indene-1,3(2H)-dione (**3B**)
  The product was obtained as a red powder; 1.938 g (69.96%). m.p. 203–205 °C. C_18_H_15_NO_2_ (calcd 277.34). ESI-MS: (M + H)^+^ = 278.20. ^1^H-NMR (CDCl_3_), δ [ppm]: δ 8.51 (d, *J* = 10 Hz, 2H -Ar), 7.91(m, 2H -Ar), 7.77 (s, 1H =CH-), 7.70 (m, 2H -Ar), 6.73 (d, *J* = 10 Hz, 2H -Ar), 3.13 (s, 6H -CH_3_). ^13^C-NMR (CDCl_3_), δ [ppm]: 191.27 (aliphatic C in ring), 190.14 (aliphatic C in ring), 154.01, 147.61 (=CH-), 142.40, 138.09, 134.54, 123.31, 122.64, 40.31 (N-CH_2_).
2-(4-methoxybenzylidene)-1H-indene-1,3(2H)-dione (**3C**)
  The product was obtained as a red/orange powder; 1.663 g (62.92%); m.p. 156–157 °C. C_17_H_12_O_3_ (calcd 264.29). ESI-MS: (M + H)^+^ = 265.15. ^1^H-NMR (CDCl_3_), δ [ppm]: δ 8.52 (d, *J* = 10 Hz, 2H -Ar), 7.96 (m, 2H -Ar), 7.82 (s, =CH-), 7.76 (m, 2H -Ar), 6.99 (m, 2H -Ar), 3.89 (s, -CH_3_). ^13^C-NMR (CDCl_3_), δ [ppm]: 190.95 (aliphatic C in ring), 189.65 (aliphatic C in ring), 164.20, 146.97 (=CH-), 142.53, 140.12, 137.34, 135.09, 126.67, 123.21, 114.52, 55.73 (-OCH_3_).
2-{[4-(4-methylphenoxy)phenyl]methylidene}-1H-indene-1,3(2H)-dione (**3D**)
  The product was obtained as a yellow powder; 0.518 g (60.99%); m.p. 146–147 °C. C_23_H_16_O_3_ (calcd 340.39). ESI-MS: (M + H)^+^ = 341.20. ^1^H-NMR (CDCl_3_), δ [ppm]: δ 8.54 (d, *J* = 10 Hz, 2H -Ar), 8.01 (m, 2H -Ar), 7.88 (s, =CH-), 7.83 (m, 2H -Ar), 7.25 (m, 2H -Ar), 7.05 (m, 4H -Ar), 2.4 (s, -CH_3_). ^13^C-NMR (CDCl_3_), δ [ppm]: 190.78 (aliphatic C in ring), 189.48 (aliphatic C in ring), 163.09, 152.77, 146.53 (=CH-), 142.55, 140.14, 135.29, 135.09, 134.81, 130.73, 127.74, 120.58, 117.25, 20.96 (CH_3_).
2-[(3-hydroxy-4-methoxyphenyl)methylidene]-1H-indene-1,3(2H)-dione (**3E**)
  The product was obtained as a lemon powder; 2.084 g (74.35%); m.p. 227–228 °C. C_17_H_12_O_4_ (calcd 280.29). ESI-MS: (M + H)^+^ = 281.15. ^1^H-NMR (CDCl_3_), δ [ppm]: δ 8.27 (s, 1H = CH-), 8.06 (m, 1H -Ar), 7.98 (m, 2H -Ar), 7.77 (m, 2H -Ar), 6.96 (d, *J* = 5 Hz, 1H -Ar), 5.66 (s, 1H -Ar), 3.99 (s, 3H -Ar), 3.85 (s, 1H -OH). ^13^C-NMR (CDCl_3_), δ [ppm]: 190.90 (aliphatic C in ring), 189.46 (aliphatic C in ring), 151.40, 147.28, 145.61 (=CH-), 142.63, 140.14, 135.28, 135.04, 123.31, 123.26, 110.53, 56.30 (-CH_3_).
2-[(naphthalen-1-yl)methylidene]-1H-indene-1,3(2H)-dione (**3F**)
  The product was obtained as an orange powder; 0.883 g (62.10%); m.p. 175–176 °C. C_20_H_12_O_2_ (calcd 284.32). ESI-MS: (M + H)^+^ = 285.10. ^1^H-NMR (CDCl_3_), δ [ppm]: δ 8.78 (s, =CH-), 8.74 (d, *J* = 10 Hz, 1H -Ar), 8.24 (d, *J* = **1**0 Hz, 1H -Ar), 8.02 (m, 3H -Ar), 7.91 (d, *J* = 5 Hz, 1H -Ar), 7.81 (m, 2H -Ar), 7.62 (m, 2H -Ar), 7.55 (t, *J* = 5 Hz, 1H -Ar). ^13^C-NMR (CDCl_3_), δ [ppm]: 190.32 (aliphatic C in ring), 188.81 (aliphatic C in ring), 143.22, 142.69, 140.23 (=CH-), 135.60, 135.43, 133.73, 133.66, 132.89, 132.22, 130.24, 129.29, 128.76, 127.80, 126.48, 125.30, 123.76, 123.53.
2-[(4,7-dimethoxynaphthalen-1-yl)methylidene]-1H-indene-1,3(2H)-dione (**3G**)
  The product was obtained as a brown-beige powder; 1.305 g (37.89%); m.p. 223–225 °C. C_22_H_16_O_4_ (calcd 344.38). ESI-MS: (M + H)^+^ = 345.15. ^1^H-NMR (CDCl_3_), δ [ppm]: δ 9.20 (d, *J* = 5 Hz, 1H -Ar), 8.68 (s, 1H =CH-), 8.24 (d, *J* = 10 Hz, 1H -Ar), 7.99 (m, 2H -Ar), 7.77 (m, 2H -Ar), 7.54 (s, 1H -Ar), 7.15 (d, *J* = 5 Hz, 1H -Ar), 6.87 (d, *J* = 5 Hz, 1H -Ar), 4.09 (s, 3H -CH_3_), 4.00 (s, 3H -CH_3_). ^13^C-NMR (CDCl_3_), δ [ppm]: 191.31 (aliphatic C in ring), 189.49 (aliphatic C in ring), 161.23, 160.06, 143.04, 142.53 (=CH-), 140.00, 136.83, 135.12, 134.90, 126.69, 125.08, 123.15, 123.07, 120.86, 120.56, 117.71, 102.69, 56.12 (-OCH_3_), 55.67 (-OCH_3_).
2-[(furan-3-yl)methylidene]-1H-indene-1,3(2H)-dione (**3H**)
  The product was obtained as a brown powder; 0.612 g (27.30%); m.p. 152–153 °C. C_14_H_8_O_3_ (calcd 224.22). ESI-MS: (M + H)^+^ = 225.10. ^1^H-NMR (CDCl_3_), δ [ppm]: δ 8.64 (s, 1H =CH-), 7.97 (m, 2H -Ar), 7.78 (m, 2H -Ar), 7.72 (s, 1H furan ring), 7.52 (m, 1H furan ring), 7.38 (m, 1H furan ring). ^13^C-NMR (CDCl_3_), δ [ppm]: 190.34 (aliphatic C in ring), 189.53 (aliphatic C in ring), 152.45, 144.53, 142.48, 140.40, 135.40, 135.18 (=CH-), 135.00, 127.76, 123.39, 123.24, 121.99, 113.08.
2-{[4-(diphenylamino)phenyl]methylidene}-1H-indene-1,3(2H)-dione (**3I**)
  The product was obtained as a brick-red powder; 1.617 g (80.57%); m.p. 211–212 °C. C_28_H_19_NO_2_ (calcd 401.48). ESI-MS: (M + H)^+^ = 402.20. ^1^H-NMR (CDCl_3_), δ [ppm]: δ 8.40 (d, *J* = 10 Hz, 2H -Ar), 7.96 (m, 2H -Ar), 7.80 (s, 1H =CH-), 7.77 (m, 2H -Ar), 7.38 (m, 2 × 2H -Ar), 7.22 (m, 2 × 3H -Ar), 7.03 (d, *J* = 10 Hz, 2H -Ar). ^13^C-NMR (CDCl_3_), δ [ppm]: 191.41 (aliphatic C in ring), 189.79 (aliphatic C in ring), 152.87, 146.73, 145.91, 142.52, 140.13, 136.95, 134.92, 134.68, 129.90, 126.71, 125.65, 122.99, 122.96, 119.02.
2-[(4-hydroxyphenyl)methylidene]-1H-indene-1,3(2H)-dione (**3J**)
  The product was obtained as a red powder; 0.980 g (78.40%); m.p. 238–240 °C. C_16_H_10_O_3_ (calcd 250.26). ESI-MS: (M + H)^+^ = 251.10. ^1^H-NMR (DMSO-d6), δ [ppm]: δ 6.94 (d, *J* = 14 Hz, 2H -Ar); 7.74 (s, 1H =CH); 7.92 (m, 4H -Ar); 8.53 (d, *J* = 14 Hz, 2H -Ar); 10.91 (s, 1H -OH). ^13^C-NMR (DMSO-d6), δ [ppm]: 189.98 (aliphatic C in ring), 189.02 (aliphatic C in ring), 163.31, 146.30 (=CH-), 141.66, 137.60, 135.55, 135.38, 122.75, 122,70, 116.00.
2-[(6-hydroxy-2H-1-benzopyran-3-yl)methylidene]-1H-indene-1,3(2H)-dione (**3K**)
  The product was obtained as a brown-black powder; 0.116 g (15.24%); m.p. > 260 °C. C_19_H_12_O_4_ (calcd 304.31). ESI-MS: (M + H)^+^ = 305.1. ^1^H-NMR (DMSO-d6), δ [ppm]: δ 5.33 (s, 2H); 6.70 (s, 1H -Ar); 6.76 (d, *J* = 1.4 Hz, 2H -Ar); 7.40 (s, 1H -Ar); 7.71 (s, 1H =CH); 7.94 (m, 4H -Ar), 9.31 (s, 1H -OH). ^13^C-NMR (DMSO-d6), δ [ppm]: 189.35 (aliphatic C in ring), 188.55 (aliphatic C in ring), 152.05, 148.25, 142.62 (=CH-), 140.86, 135.79, 130.08, 122.93, 122.85, 116.58, 114.34, 66.55.
